# GANAB as a Novel Biomarker in Multiple Sclerosis: Correlation with Neuroinflammation and IFI35

**DOI:** 10.3390/ph14111195

**Published:** 2021-11-21

**Authors:** Roberto De Masi, Stefania Orlando

**Affiliations:** 1 Complex Operative Unit of Neurology, “F. Ferrari” Hospital, Casarano, 73042 Lecce, Italy; dmsrrt@gmail.com; 2 Laboratory of Neuroproteomics, Multiple Sclerosis Centre, “F. Ferrari” Hospital, Casarano, 73042 Lecce, Italy

**Keywords:** GANAB, IFI35, neuroinflammation, multiple sclerosis, interferon

## Abstract

Multiple sclerosis (MS) still lacks reliable biomarkers of neuroinflammation predictive for disease activity and treatment response. Thus, in a prospective study we assessed 55 MS patients (28 interferon (IFN)-treated, 10 treated with no-IFN therapies, 17 untreated) and 20 matched healthy controls (HCs) for the putative correlation of the densitometric expression of glucosidase II alpha subunit (GANAB) with clinical/paraclinical parameters and with interferon-induced protein 35 (IFI35). We also assessed the disease progression in terms of the Rio Score (RS) in order to distinguish the responder patients to IFN therapy (RS = 0) from the non-responder ones (RS ≥ 1). We found GANAB to be 2.51-fold downregulated in the IFN-treated group with respect to the untreated one (*p* < 0.0001) and 3.39-fold downregulated in responder patients compared to the non-responders (*p* < 0.0001). GANAB correlated directly with RS (r = 0.8088, *p* < 0.0001) and lesion load (LL) (r = 0.5824, *p* = 0.0014) in the IFN-treated group and inversely with disease duration (DD) (r = −0.6081, *p* = 0.0096) in the untreated one. Lower mean values were expressed for GANAB than IFI35 in IFN responder (*p* < 0.0001) and higher mean values in the non-responder patients (*p* = 0.0022). Inverse correlations were also expressed with IFI35 in the overall patient population (r = −0.6468, *p* < 0.0001). In conclusion, the modular expression of GANAB reflects IFI35, RS, DD, and LL values, making it a biomarker of neuroinflammation that is predictive for disease activity and treatment response in MS.

## 1. Introduction

Multiple sclerosis (MS) is a degenerative and inflammatory chronic disease that affect the central nervous system (CNS). Despite the many studies suggesting the critical role of peripheral autoreactive T-cells in the demyelination process and axonal loss [1], there is still no usable cell-based biomarker of disease activity [2,3]. Interferon-beta (IFNbeta) is a disease-modifying therapy (DMT) that reduces neuroinflammation in relapsing-remitting (RR) MS, acting on peripheral blood mononuclear cells (PBMCs) with an MRI-detectable effect, confirming the critical role of PBMCs in the CNS damage of disease subjects [4]. However, this drug is not always effective and there are no relevant markers to predict the response to it yet. The Rio Score (RS) or Modified Rio Score (MRS) are the only currently available tools as clinical predictors of treatment response to IFNbeta [5]. However, they are very difficult to manage due to the clinical/paraclinical setting and the long assessment time (more than one year) required, during which severe disabilities can develop. In this scenario, the identification of predictive markers of response to therapy would make it possible to avoid emerging disability in MS patients. In previous two-dimensional electrophoresis studies, some differentially expressed proteins (DEPs) have been highlighted in PBMCs from IFN-treated MS patients in comparison to untreated ones as well as healthy controls (HCs) [6,7]. These DEPs include interferon induced protein 35 (IFI35, also called IFP35) and glucosidase II alpha subunit (GANAB). While the former has recently been found to be an indicator of innate immunity-dependent neuroinflammation and clinical progression in MS [8], the properties of the latter still remain under investigation. However, the role of glycosylation in the maturation process of key proteins of both innate and adaptive immune responses has already been described [9,10]. In fact, several studies have shown the altered glycosylation process to be linked to an increased susceptibility to developing MS through lymphocytic dysfunction [11,12,13]. This context also includes the role of vitamin D3, which inhibits T lymphocyte activation and differentiation into Th1 by regulating their N-glycosylation [14]. Moreover, GANAB is involved in the endoplasmic reticulum (ER) stress response (ERSR), also called unfolded protein response (UPR) [15,16]. This is a mechanism of recovery from protein unfolding/misfolding within the ER that is induced by chronic inflammatory conditions and results in the activation of several enzymes and chaperones, including GANAB, calnexin, and reticulin. This leads to proteostatic achievement by enhancing the degradation of mRNAs via IRE1-dependent decay [17,18].

In effect, GANAB is a heterodimeric enzyme that is involved in the glycosylation of N-glycans in post-translational protein modification in the ER. This glycoenzyme interacts with CD45 through the lectin-dependent mannose pathway. CD45 is a heavily glycosylated transmembrane tyrosine-phosphatase that is abundantly expressed in all nucleated cells of hematopoietic origin (constituting about 10% of the total surface proteins) [19]. It has been shown that CD45 activity is essential in the cascade events of signal transduction, leading to thymocyte maturation and T cell activation [20]. Some authors have suggested that the glycosidic structure of CD45 changes as T cells mature and that this change mainly involves GANAB’s ability to bind CD45. These modifications of CD45 glycosylation would have important implications in various biological processes, such as the development of the plasma membrane, vesicular trafficking, and cell adhesion [21].

GANAB participates in the superpathway of the calnexin–calreticulin cycle. Calnexin and its luminal homolog calreticulin are two membrane-bound chaperones that are involved in the mechanism control of protein folding; they require a monoglycosylated glycan to bind proteins in their maturation phase [22], and this glucose trimming is carried out by GANAB. The involvement of N-glycans in the ER “quality control” of correct protein folding (ERQC, ER Quality Control Compartment) explains the key role of this kind of glycosylation and also suggests why defects in the proteins involved in these reactions are frequently associated with congenital polycystic diseases. Recently, some authors have suggested that calnexin is involved in the transmigration of T lymphocytes within the CNS, showing the chaperon to be highly expressed in endothelial cells of the blood–brain barrier (BBB) of MS patients and demonstrating that knockout mice for calnexin are resistant to the induction of experimental autoimmune encephalomyelitis (EAE) (i.e., the MS animal model) [23]. Furthermore, other studies have shown the overexpression of GANAB in the Th1 cells of patients with lupus erythematosus in the active stage of the disease [24]. Finally, the UPR is activated in oligodendrocytes, T cells, macrophages/microglia, and astrocytes, as well as regulating the viability in oligodendrocyte and axons of MS patients and EAE mice model [25,26,27].

The primary aim of our study is to test GANAB for putative clinical relevance in MS. For this purpose, the predictive value of the densitometric expression of GANAB from PBMCs with respect to neuroinflammation was assessed in IFN-treated and untreated MS patients compared to HCs. Specifically, we statistically correlated GANAB with the clinical and paraclinical parameters of disease subjects. Furthermore, we aimed to assess the modular expression of GANAB with RS and MRS rank in order to identify a risk value of clinical progression or unfavorable clinical outcome for each IFN-treated MS patient.

Finally, we studied the quantitative correlation between GANAB and IFI35 in the overall MS study population. The IFI35 expression profile, in fact, is already known to be correlated with RS and MRS rank values, white matter volume, and brain lesion load (LL), representing an emerging marker of neuroinflammation in MS [8].

## 2. Results

**Figure 1:**
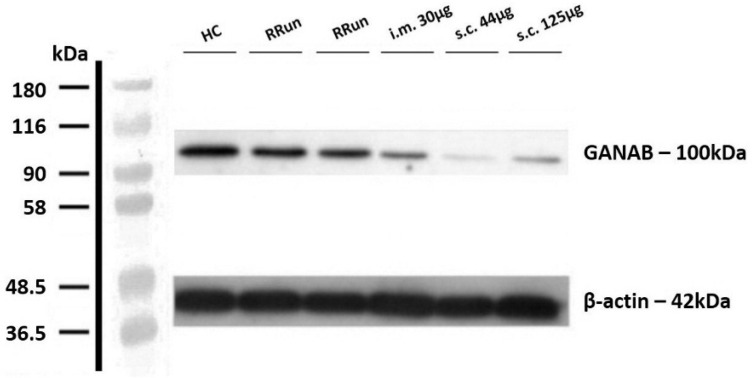
Example image that representing the western blotting of GANAB. Value of GANAB increasing from the IFNbeta-1a-treated MS patients (intramuscular injection (i.m.) 30 μg, subcutaneous injection (s.c.) 125 μg, s.c. 44 μg) to relapsing remitting untreated patients (RRun) compared to HCs. Note the gradually decreasing densitometry from the intramuscular to the subcutaneous formulation. All densitometric measurements of GANAB were normalized with beta-actin. HC, healthy control; RRun, relapsing remitting untreated patient; i.m. 30 μg, patient treated with a 30 μg intramuscular weekly formulation of IFNbeta-1a; s.c. 44 μg, patient treated with the subcutaneous high-dose three-times weekly formulation of IFNbeta-1a; s.c. 125 μg, patient treated with the subcutaneous pegylated formulation of IFNbeta-1a every two weeks.

We analyzed the densitometric expression of GANAB for the entire study population, based on the immunoblotting technique. The normalized value of GANAB resulted from the ratio between the optic densitometry of GANAB and the beta-actin one. Figure 1 shows the modular expression of GANAB.

The clinical and demographic characteristics of the compared groups are shown in Table 1. Specifically, the HCs had mean age of 45.07 ± 12.09 years without any significant differences from relapsing remitting untreated patients (RRun), IFNbeta-1a, and other therapy (other th.) treated patients expressing, respectively, 46.06 ± 11.79, 46.84 ± 10.74, and 44.2 ± 6.22 years. The mean disease duration (DD) was 15.75 ± 10.08, 14.48 ± 8.23, and 12.9 ± 6.13 years, respectively, for RRun, IFNbeta-1a, and other therapies. The mean Expanded Disability Status Scale (EDSS) score was 1.84 ± 1.03, 1.95 ± 1.35, and 2.02 ± 0.57, respectively, for RRun, IFNbeta-1a, and other therapies.

**Figure 2:**
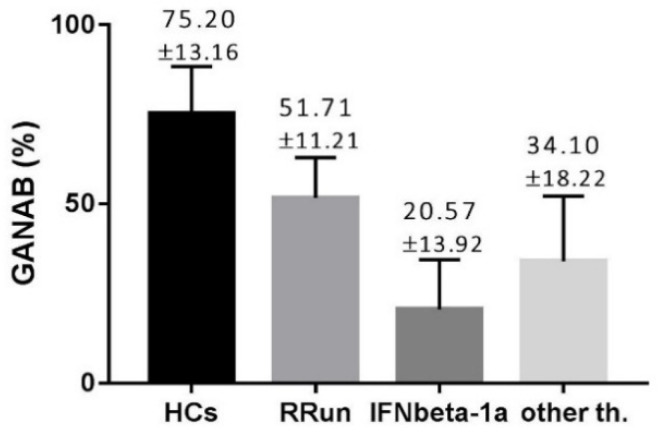
The modular expression of GANAB in all compared groups. HCs, control group; RRun, relapsing remitting untreated MS patients; IFNbeta-1a, MS patients treated with IFNbeta-1a; other th., MS patients treated with other than IFN treatment. Values are shown as percentages means with standard deviation, normalized on beta-actin.

**Figure 3:**
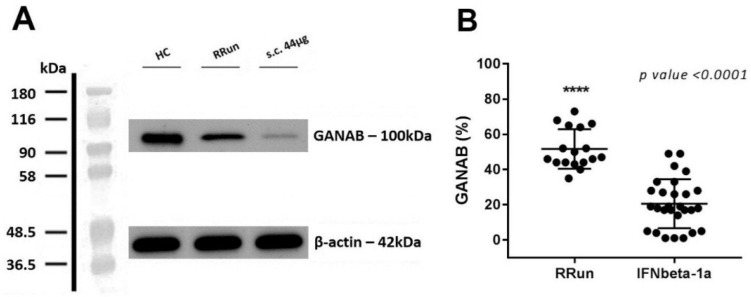
GANAB expression in IFNbeta-1a-treated and RRun MS groups. (**A**) Western blotting image for GANAB from one IFN-treated patient that underwent the s.c. 44 μg of IFNbeta-1a formulation (s.c. 44 μg), from one untreated relapsing remitting patient (RRun), and finally from one healthy control (HC). (**B**) Comparison between GANAB expression from the relapsing remitting untreated MS group (RRun) and the IFNbeta-1a-treated MS group (IFNbeta-1a). The expression of GANAB is upregulated 2.51-fold in the untreated with respect to the treated MS group.

The GANAB values, shown as volume percentage normalized on beta-actin, were 72.20 ± 13.16, 51.71 ± 11.21, 20.57 ± 13.92, and 34.10 ± 18.22%, respectively, for HCs, RRun, IFNbeta-1a, and the other therapy groups. These values are graphically represented in Figure 2. We found the main GANAB differential expression by comparing the RRun group and the IFNbeta-1a-treated patients (*p* < 0.0001). Figure 3 shows this comparison with a representative western blotting image.

**Figure 4:**
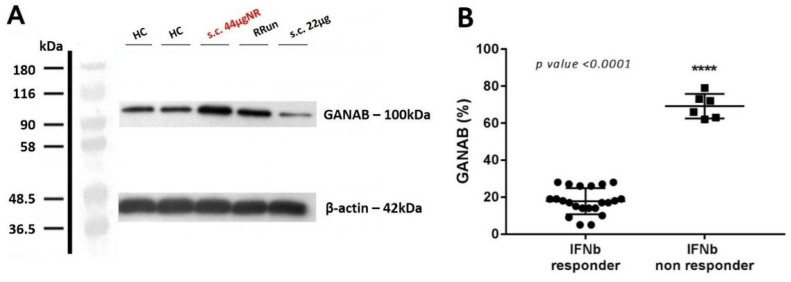
GANAB in the IFNbeta responder and non-responder patients. (**A**) Representative image of Western blotting for GANAB from one non-responder patient that received s.c. 44 μg of IFNbeta-1a formulation (s.c. 44 μgNR) (red). GANAB expression is represented for healthy controls (HCs), for one untreated relapsing remitting patient (RRun), and finally from a responder IFN-treated patient who underwent the s.c. 22 μg formulation (s.c. 22 μg). Note the upregulation of GANAB in the non-responder as well in the untreated patient compared to the responder. (**B**) Comparison of the mean expression of GANAB (%) between the responder and non-responder IFN groups.

Furthermore, for the modularity of GANAB we noted the clinical progression accordingly. Specifically, we found GANAB expression to be statistically higher by 3.39-fold in the non-responder (69.17%) compared to responder (17.82%) patients (*p* < 0.0001). Figure 4A shows an example of one patient with a strongly up-regulated GANAB expression, despite the high-dose interferon therapy applied. A total of six non-responder patients were analyzed. Figure 4B shows the mean expression of GANAB in the responder compared to the non-responder patients.

**Figure 5:**
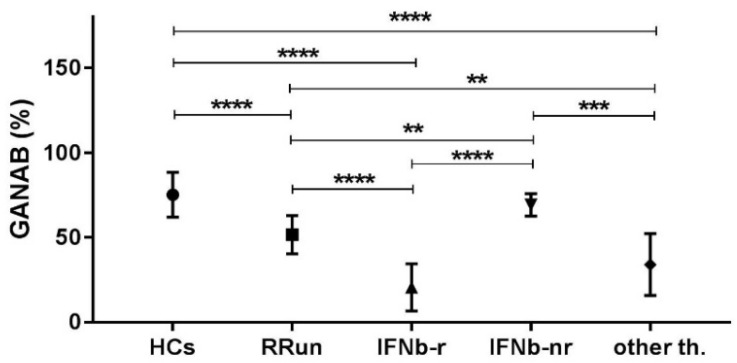
Comparison of the mean expression of GANAB (%) in each group considered in the study. Symbol meaning: ** = *p* < 0.01; *** = *p* < 0.001; **** = *p* < 0.0001.

By statistically comparing the GANAB values from all the study groups, we found significant ratios (expressing how many times a molecule is up- or down-regulated in one group compared to another) ≥2 in the following comparisons: HCs/IFNbeta responder; RRun/IFNbeta responder; IFNbeta non-responder/IFNbeta responder; HCs/other therapies and other therapies/IFNbeta non responder. The significant ratios non-reaching value of 2 we found for the following comparison: HCs/RRun; RRun/other therapies and IFNbeta non responder/RRun. These data are detailed in Figure 5 and relative ratios and *p* values are summarized in Table 2.

**Figure 6:**
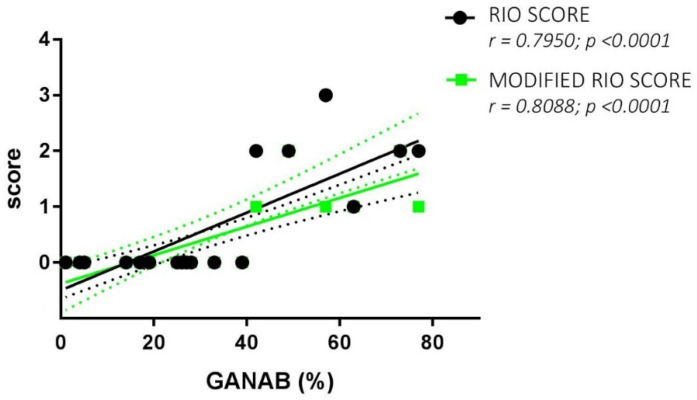
GANAB and RS/MRS correlations. Direct significant correlation between GANAB (%) and Rio Score (green)/Modified Rio Score (black) for the entire cohort of IFN-treated MS patients. Regression lines are shown with the relative standard deviation as dotted lines of the same color.

**Figure 7:**
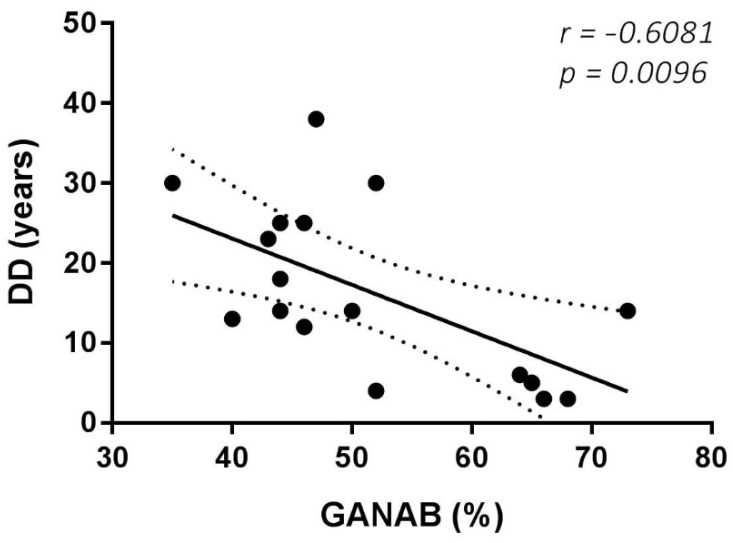
GANAB and DD correlation. Significant inverse correlation between GANAB (%) and DD (disease duration in years) in the untreated MS group.

The Spearman rank test evidenced good correlations between both the RS and MRS scores and the GANAB densitometric expression (r = 0.7950, *p* < 0.0001; r = 0.8088, *p* < 0.0001, respectively). Figure 6 shows the correlation of GANAB with the RS/MRS values. Furthermore, we found an inverse significant correlation between GANAB expression and the DD in the RR untreated MS group (r = −0.6081, *p* = 0.0096), as shown in Figure 7.

**Figure 8:**
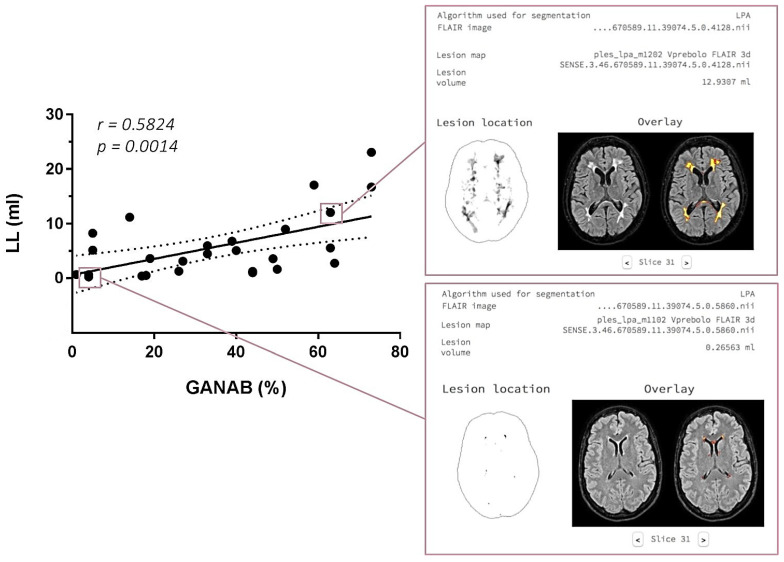
On the left: GANAB and LL correlation. Significant direct correlation between the quantitative expression of GANAB (%) and lesion load (LL) expressed in ml in the IFN-treated MS group. On the right: output image in the LPA report with segmented lesions on the 3DFLAIR image of two IFN-treated MS patients, one with a higher GANAB expression (63%) and lesion volume (12.9307 mL) (up) compared to the other with a lower GANAB expression (4%) and lesion volume (0.2656 mL) (down).

With regard to the MRI post-analysis, the logistic regression analysis evidenced a significant correlation between the GANAB expression and LL in the IFN-treated MS group, with r = 0.5824 and *p* = 0.0014. This correlation and an example of the LPS output image are represented in Figure 8.

Through the cortical and sub-cortical parcellation in the IFN-treated patients, we evidenced significant correlations between several segmented brain regions and GANAB expression after Bonferroni correction for multiple comparisons was performed. These correlated brain regions are represented in Table 3, highlighting the 4th ventricle volume (*p* = 0.0422), left nucleus accumbens (*p* = 0.0240), right cerebellum white matter volume (*p* = 0.0200), and total estimated intracranial volume (*p* = 0.0057).

**Figure 9:**
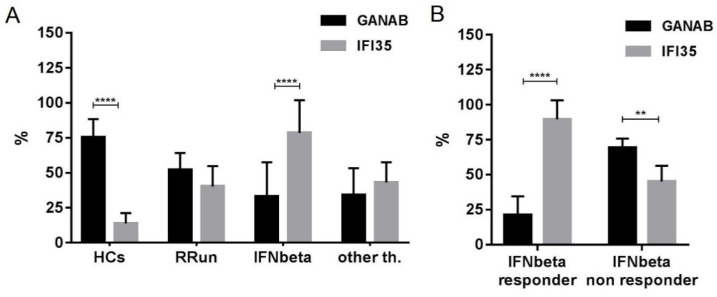
Expression of GANAB and IFI35 in all enrolled groups. Note statistically significant differences between the means of GANAB and IFI35 in (**A**) the HCs (*p* < 0.0001) and IFNbeta-treated patients (*p* < 0.0001), as well as in (**B**) the IFNbeta responder (*p* < 0.0001) and IFNbeta non-responder (*p* = 0.0022) patients. HCs, healthy controls; RRun, relapsing remitting untreated MS patients; IFNbeta responder, responder MS patients to IFNbeta-1a therapies; IFNbeta non responder, non responder MS patients to IFNbeta-1a therapies; other th., MS patients treated with other than interferon therapies. Symbol meaning: ** = *p* < 0.01; **** = *p* < 0.0001.

**Figure 10:**
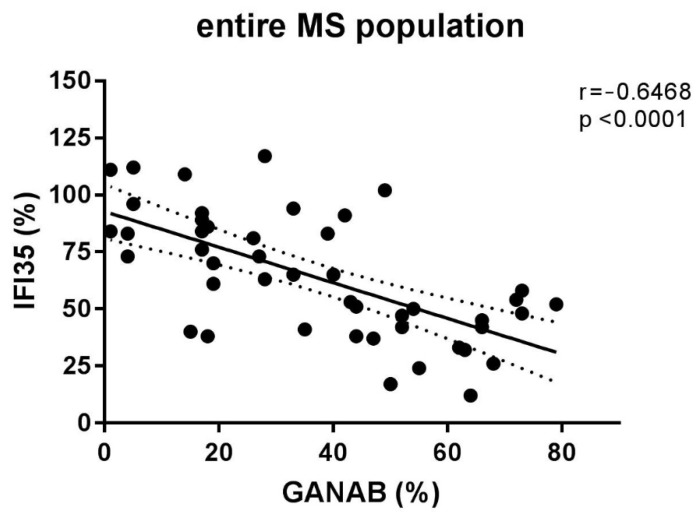
Correlation between the densitometric expression of GANAB and IFI35 in the entire MS population.

**Figure 11:**
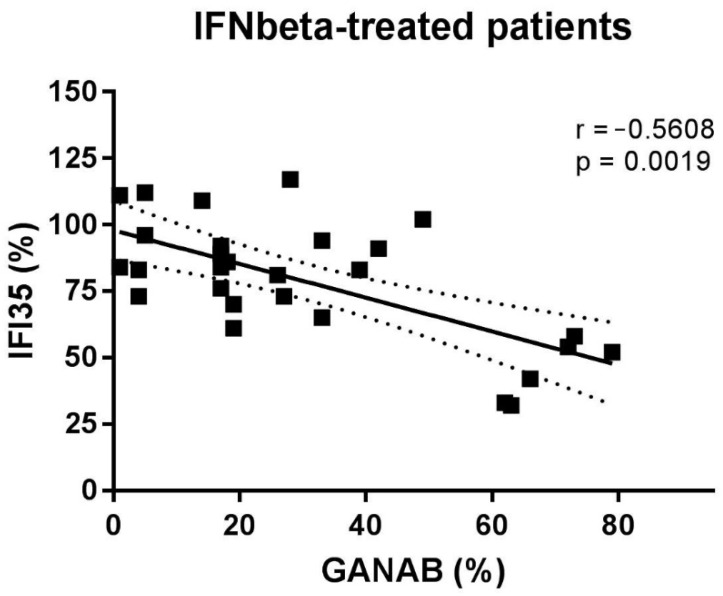
Correlation between GANAB and IFI35 in IFNbeta-treated patients.

The densitometric expression of GANAB for each enrolled subject was also correlated with that of IFI35 from the same blood sample and with the same methods described in a previous study. From the comparison between all the enrolled groups, we found statistically significant differences between the means of the GANAB values and the IFI35 ones in both the HCs (*p* < 0.0001) and IFN-treated groups (*p* < 0.0001) (Figure 9A). Specifically, in the latter group, we also found statistically significant differences in the IFN-treated responder (*p* < 0.0001) and non-responder patients (*p* = 0.0022), with two opposite molecular modulation patterns for these two proteins (Figure 9B). Furthermore, the Spearman rank test evidenced significant inverse correlations between GANAB and IFI35 not only in the entire study cohort of MS patients (r = −0.6468, *p* < 0.0001) (Figure 10) but also in the IFN-treated group (r = −0.5608, *p* = 0.0019) (Figure 11).

## 3. Discussion

The availability of reliable biomarkers could radically change the management of MS. In fact, predictive markers of disease activity or therapeutic efficacy would allow intervention strategies able to prevent the disease’s progression or to establish an ineffective therapy before the disability accumulation in each patient. These unmet needs are also evident in the interferon treatment for MS.

Despite worldwide scientific efforts, MS still remains a pathology with unknown etiology. Currently no etiological marker is available and the diagnosis is based uniquely on the physiopathological concept of dissemination in space (DIS) and time (DIT) of the CNS lesions. Not by chance, current McDonald revised criteria [28] emphasizing the morphology and the location of typical lesions than their quantity, consider these as diagnostic paraclinical marker of DIS and DIT. The latter is also assessed by the intrathecal synthesis of oligoclonal bands (OCB) [28]. In fact, OCB reflect the clonal expansion over time of immunoglobulin-secreting B cells in the ectopic subpial lymphoid follicles.

Here, we aimed to assess the use of PBMCs for GANAB regulation for predicting neuroinflammation in diseased subjects already diagnosed with MS. With this purpose, a comparative, clinical/paraclinical, and molecular prospective study was performed.

Published studies lack reports on GANAB involvement in CNS pathologies, apart from our proteomic observation carried out in 2009 [7]. The existing articles mention it only with regard to the polycystic condition of the kidney and liver due to the autosomal mutation of its gene. Only recently have several works indicated ER stress to be linked to MS pathology and human autoimmune chronic inflammatory diseases. GANAB is a well-known regulating factor of this process and is also correlated to the UPR [29,30]. With regard to the IFI35, this is a biomolecular marker of neuroinflammation that has been actively studied in our group and in the Xiahou one [31] independently. In nature, it serves as a molecule of damage-associated molecular patter (DAMP) via Toll-like receptors.

In the present study, we demonstrated the modular expression of GANAB, firstly highlighting how interferon therapy downregulates it. Specifically, we measured an expression that was 2.51-fold lower (*p* < 0.0001) in IFNbeta-treated patients compared to that in untreated ones. The expression of the molecule can also be reduced by treatments other than interferon, albeit in a less effective way. We measured a 1.51-fold reduction (*p* = 0.0043) in the GANAB expression from the group undergoing DMT other than IFNbeta in comparison to the untreated one.

From the relationship analysis carried out between GANAB and clinical variables, an interesting result emerged in the untreated group: an inverse correlation between GANAB expression and DD. A plausible explanation of this finding is the immunosenescent phenomenon, which has already been described for MS and other organ-specific chronic inflammatory diseases, resulting in a reduction in glycoenzyme activity over the disease’s natural course. This correlation with a time-dependent variable can explain the lower GANAB expression in the RRun group compared to the HC one. In effect, our RRun study population expressed an average DD value of 15.75 ± 10.08, given the similar mean age between two groups. Consistently, the importance of age as a biological variable influencing the natural course of the disease and its response to therapy is an emerging topic in research on MS and other chronic inflammatory human diseases [32,33].

In addition, even lower average values were found for GANAB expression in IFN-treated MS patients if they were responders to therapy. In particular, the expression profile was 3.39-fold higher (*p* < 0.0001) in the non-responder group than in the responder group. This finding confirms the modularity of the expression of GANAB, with values from the untreated subjects differing significantly compared to those of the IFN-treated ones, as well as from the effectively treated patients to the ineffectively treated ones. This molecular profile of GANAB suggests that it is a biologically relevant element for MS and sensitive not only to the disease but also to its response to therapy. In effect, mainly the interferon as well as other therapies downregulated this molecule. However, if the drug is not effective, an increase in the inflammatory condition will follow the increase in the expression of GANAB. These deductions are confirmed by the correlation between the GANAB values and MRS/RS (*p* < 0.0001 in both cases). This direct correlation also further confirms that MS patients expressing high GANAB levels belong to the group of patients treated with IFN who have an RS and MRS rank ≥1 (Figure 6) and show disease progression during a one-year observation period after their enrolment.

Consistently, we found a direct statistical correlation between GANAB expression and LL (r = 0.5824; *p* = 0.0014) in the IFN-treated patients, confirming again that the molecular regulation reflects the efficacy of IFN in reducing disease activity and neuroinflammation, as expressed by total brain LL. Additionally, the direct correlation of the fourth ventricle amplitude as well as the inverse one of the right cerebellum white matter volume and left area of the nucleus accumbens with GANAB expression highlights its predictive ability with respect to brain atrophy as a common final of neuroinflammation in MS.

We also found significant differences between the mean expression values of GANAB in comparison to IFI35 in both the IFN-treated responder and non-responder groups.

Based on this difference, we determined a responder pattern for cases of patients who had undergone effective interferon therapy resulting in downregulated GANAB and upregulated IFI35 expression, as well as a non-responder pattern in cases of patients who experience an increase in inflammatory conditions due to the failure of interferon treatment, resulting in increased GANAB and decreased IFI35 expression. Specifically, the significant direct correlation between RS/MRS and GANAB as well as the inverse one with IFI35 confirms once more that MS patients expressing high GANAB and low IFI35 values belong to the group of patients who experienced disease progression. In addition, the low expression of GANAB and high expression of IFI35 reflect the ability of interferon activity to reduce the lesion burden. These findings describe a molecular panel that, although not yet part of the clinical routine, adds relevant information about the physiopathology of MS.

In effect, we also found GANAB and IFI35 to be inversely correlating factors across the entire diseased population. This interesting result does not exactly suggest the existence of interplaying functions based on a common molecular pathway or multicomponent metabolic machinery involving these chemical species, such as their common sensitivity to MS-related neuroinflammation. In fact, it results more from our observations of a characteristic *continuum* ranging from untreated patients to the non-responder ones and finally to the responder. Specifically, in the IFN-treated group, a possible explanation for the inverse correlation between the densitometric expression of GANAB and IFI35 derives from the IFN-dependent suppression effect on protein synthesis and cell proliferation. This is a highly conserved process, evolutionarily acting from fish to humans and resulting in a homeostatic anti-inflammatory/anti-proliferative response. This protective effect of IFN was exploited for therapeutic purposes in MS but also involves GANAB, according to our data, which acted as expected as a sensor molecule to neuroinflammation.

In conclusion, we found GANAB to be a reliable biomarker for MS, with it being predictive not only for the response to DMT and disease course in IFN-treated subjects but also for disease activity linked to innate immunity-dependent neuroinflammation. A limitation of this study is the sample size used, which, although small, does not reduce the reliability of the conclusions, as it confirms and extends the results of our preliminary studies on this topic.

## 4. Materials and Methods

### 4.1. Study Design

In a comparative, clinical/paraclinical, and molecular prospective study, we enrolled 55 IFN-treated and untreated MS patients consecutive and unselected for age, sex, or ethnicity. All these attended the Multiple Sclerosis Centre of Neurological Department at the “F. Ferrari” Hospital in Casarano, Lecce (Italy). A comparison group of 20 healthy controls was also considered.

Each enrolled subject underwent blood sampling at the study entry and the value of GANAB was calculated. In addition, brain MRIs were performed on patients at their time of entry and they were enrolled in a three-year follow-up program. The latter involved a neurological examination every three months as well as an MRI once a year; other evaluations assessed the RS and MRS rank instead. Closest to time withdrawal, a multi-sequence MRI imaging study that was T1- and T2-weighted (w), fluid inversion recovery (FLAIR), three-dimensional (3D) T1w, and 3DFLAIR acquisition modality studies were performed to assess the brain atrophy in each patient. The RS and MRS rank and GANAB expression of each enrolled MS patient were correlated with each other to investigate the predictive profile of GANAB with respect to therapeutic response to IFN. We considered the patients with RS and MRS = 0 to be responders and the patients with RS or MRS ≥ 1 to be non-responders.

### 4.2. Study Population

The subjects’ enrollment took place at the MS Centre of Casarano during routine visits, according to the following inclusion/exclusion criteria.

The inclusion criteria consisted of untreated MS: 17 relapsing remitting untreated patients. These patients underwent no therapy since they were in the early phase of the disease or in a wash-out period from the drug.

IFN-treated MS: 28 MS patients treated with IFNbeta-1a. Specifically, 7 patients were given a 30 μg intramuscular injection (i.m.) weekly formulation, 4 underwent a 125 μg subcutaneous injection (s.c.) every two weeks in a pegylated formulation, 8 were given a 22 μg s.c. three times weekly formulation and 9 were given a 44 μg s.c. three times weekly formulation. All of these patients were Nabs negative and were also relapse- and corticosteroid-free by at least three months. All these patients had been on therapy for at least one year at the study entry point to ensure that each participant had full drug clinical activity.

MS treated with therapies other than IFN DMT: 10 MS patients treated with no-IFN therapies, including Rituximab, Dimethyl Fumarate, Fingolimod, and Natalizumab.

Healthy controls: 20 healthy subjects sex-matched with MS patients and without kinship relations with MS patients.

All MS patients were previously diagnosed according to the 2017 McDonald revised criteria [28] and examined/imaged in an exacerbation-free period of at least three months. The study was conducted according to the guidelines of the Declaration of Helsinki and approved by the Local Ethics Committee of A.S.L. LE (project ID 1057/DS of 12/10/2016). All enrolled subjects gave written informed consent for their enrollment in the study, the storage of their data, and the future use of their blood samples for research purposes.

The exclusion criteria were any metabolic, cardiovascular, or immunological comorbidity, as well transient inflammatory and septic conditions.

### 4.3. PBMC Separation and Protein Extraction

PBMC separation and protein extraction were carried out as previously described [8]. Briefly, after venipuncture, 16 mL of heparinized blood was diluted with 1:1 Phosphate Buffered Saline solution (PBS 1X) and layered on a density gradient of Ficoll–Hypaque (GE Healthcare). From the obtained PBMCs ring by centrifugation, proteins were extracted using a urea/thiourea buffer and their concentration was determined by the Bradford assay.

### 4.4. Electrophoresis and Western Blotting

Electrophoresis and western blotting were carried out as previously described [8]. Briefly, the electrophoretic run of PBMCs protein sample, under SDS-PAGE conditions, took place on a gradient polyacrylamide gel well (Amersham ECL Gel, GE Healthcare Life Sciences), with a pre-run current. A pre-stained molecular marker (Sigma Aldrich, St. Louis, MO, USA) was used for checking the run.

After the electrophoretic migration, proteins were transferred to a nitrocellulose membrane using classical electro-blotting “sandwich” method. Then, a blocking solution with non-fat dry milk (BIORAD) was used to block non-specific binding sites.

Subsequently, the membrane was incubated overnight at 4 °C with monoclonal anti-human GANAB antibody produced in mouse (Sigma Aldrich) (diluted 1:500 in PBS-T) and then with secondary antibody anti-mouse IgG peroxidase conjugated produced in rabbit (Sigma Aldrich) (diluted 1:60,000 in PBS-T) for 1 h at room temperature. To obtain normalized densitometric values of GANAB, we probed the membrane with anti-beta-actin antibody produced in mouse (Sigma Aldrich) (diluted 1:10,000 in PBS-T).

Chemilumiscence kits (Cyanagen) were used for the detection of proteins and photographic films for the protein bands impression. For these last, the optical densitometry was calculated using the ImageJ software. The operator was blinded with regard to the kind of sample.

### 4.5. Chemicals

Phosphate Buffered Saline 10X (BR-1006-72), Ficoll–Hypaque density gradient (17-1440-03), ECL Gel 8–16% (28-9898-07) were purchased from GE Healthcare Life Sciences AB, Uppsala, Sweden. Protease inhibitor cocktail (P8340), Pre-stained molecular weight marker (SDS7B2), Anti-GANAB antibody produced in mouse (SAB1401584), Monoclonal Anti-Actin antibody produced in mouse (A3853), Anti-mouse IgG peroxidase conjugated produced in rabbit (A9044) were obtained from Sigma Aldrich, Milan, Italy. Non-fat dry milk Blotting-Grade Bocker (170-6404) and Bio-Rad Protein Assay (500-0006) were obtained from Bio-Rad Laboratories, Milan, Italy. Westarn Nova 2.0 (XLS10), EtaC (XLS070), EtaC Ultra (XL075) chemiluminescent kits were purchased from Cyanagen, Bologna, Italy.

### 4.6. MRI Protocol

MRI scans were carried out as previously described [8]. Briefly, the MR imaging of the MS patients was performed on a 1.5-T Philips MR apparatus (180 mT/m) (Achieva, Philips Medical Systems, Best, Netherlands), in accordance with international guidelines [34].

The LL, global and selective brain atrophy were calculated in the MRI post-analysis phase. Specifically, the brain parenchymal fraction (BPF) was determined according to Rudick’s method [35]; peripheral grey matter (pGREY), grey matter (GM), white matter (WM), ventricular cerebrospinal fluid (vCSF), and total brain volume (TBV) were also calculated on T1-weighted images using the “Sienax” tool of the FLS package software (created by the Analysis Group, FMRIB, Oxford, UK).

On T1-weighted and FLAIR images of the brain we assessed the LL by LPA (Lesion Prediction Algorithm) algorithm of the LST (Lesion Segmentation Tool) of the SPM12 (Statistical Parametric Mapping) software (Functional Imaging Laboratory, Wellcome Trust Centre for Neuroimaging, Institute of Neurology, London, UK).

For the cortical and sub-cortical parcellation of the brain, the FreeSurfer software based on 3DT1 images was used. All the measurements are expressed in ml. Axial images (ax) were acquired from all T1- and T2-weighted sequences, while axial and three-dimensional images were acquired from FLAIR sequences.

### 4.7. Clinical Data

All clinical data of each patient, including the sex, age, EDSS, DD, and RS and MRS rank values were collected. The EDSS, which was developed by Kurtzke in 1983 [36], is a universal scale for neurologic impairment assessment in MS. This evaluation expresses a composite score from the combination of single sub-scores (pyramidal, visual, sensitive, cerebellar, etc.), resulting in the degree of disability, with a dimensionless numerical value ranging from 0 to 10. Finally, the RS and MRS rank values were assessed for each IFN-treated MS patient with the purpose to clinically define the responder and non-responder patient to the IFNbeta treatment. Specifically, for the Rio Score: the sum of MRI, relapse and EDSS criteria classifies patients as having a score of 0, 1, 2, or 3. For the Modified Rio Score: score 0 if new T2 lesions ≥ 4 and relapses = 0; score 1 if new T2 lesions ≥ 4 and relapses = 1 or new T2 lesions > 4 and relapses = 0; score 2 if new T2 lesions ≥ 4 and relapses ≥ 2 or new T2 lesions > 4 and relapses = 1; score 3 if new T2 lesions > 4 and relapses ≥ 2.

### 4.8. Statistical Analysis

Statistical analysis was performed with the GraphPad Prism7 software. For descriptive statistics, the non-parametric Wilcoxon–Mann–Whitney test was applied to examine the differences between means; for inferential statistics, the non-parametric Spearman test was used to determine the correlation between variables as well as logistic regression analysis. Finally, Bonferroni correction was applied, where appropriate, for multiple comparisons.

## Figures and Tables

**Table 1:** Mean values are represented with standard deviations. HCs, healthy controls; RRun, relapsing remitting untreated MS patients; IFNbeta-1a, patients treated with IFNbeta-1a; other th., MS patients treated with other than IFN DMT; DD, disease duration; EDSS, Expanded Disability Status Scale.

	HCs	RRun	IFNbeta-1a	Other th.
**N**	20	17	28	10
**Age (years)**	45.07 ± 12.09	46.06 ± 11.79	46.84 ± 10.74	44.2 ± 6.22
**DD (years)**		15.75 ± 10.08	14.48 ± 8.23	12.9 ± 6.13
**EDSS (score)**		1.84 ± 1.03	1.95 ± 1.35	2.02 ± 0.57

**Table 2:** Relative ratios values and *p* values of the compared study groups. HCs, healthy controls; RRun, relapsing remitting untreated MS patients; IFNb-r, responder MS patients to IFNbeta-1a therapies; IFNb-nr, non-responder MS patients to IFNbeta-1a therapies; other th., MS patients treated with other than interferon therapies. Symbol meaning: ↑ = upregulation, n.s. = *p* > 0.05.

**HCs vs. IFNb-r**	**RRun vs. IFNb-r**	**IFNb-nr vs. IFNb-r**	**other th. vs. IFNb-r**	**HCs vs. other th.**
↑ 3.65 (*p* < 0.0001)	↑ 2.51 (*p* < 0.0001)	↑ 3.39 (*p* < 0.0001)	n.s.	↑ 2.2 (*p* < 0.0001)
**HCs vs. RRun**	**RRun vs. other th.**	**IFNb-nr vs. RRun**	**HCs vs. IFNb-nr**	**other th. vs. IFNb-nr**
↑ 1.45 (*p* < 0.0001)	↑ 1.51 (*p* = 0.0043)	↑ 1.34 (*p* = 0.0018)	n.s.	↑ 2.03 (*p* = 0.0005)

**Table 3:** Significant correlations between the mean quantitative expression of GANAB and some cortical/sub-cortical segmented brain regions from the considered IFN-treated MS group. Bonferroni correction for multiple comparisons was applied.

GANAB vs.	r	*p* Value
4th Ventricle	0.8273	0.0422
Left Nucleus Accumbens	−0.8706	0.0240
Right Cerebellum White Matter	−0.8821	0.0200
Total Estimated Intracranial Volume	0.9377	0.0057

## Data Availability

Data is contained within the article.

## Abbreviations

BBB	blood–brain barrier
BPF	brain parenchymal fraction
CNS	central nervous system
DAMPs	damage-associated molecular patterns
DD	disease duration
DEP	differentially expressed protein
DMT	disease-modifying therapy
EAE	experimental autoimmune encephalomyelitis
EDSS	expanded disability status scale
ER	endoplasmic reticulum
ERSR	endoplasmic reticulum stress response
GANAB	glucosidase II alpha subunit
GM	grey matter
HCs	healthy controls
i.m.	intramuscular injection
IFI35	interferon-induced protein 35
IFN	interferon
LL	lesion load
MRS	Modified Rio Score
MS	multiple sclerosis
other th.	patients treated with other than IFN therapy
PBMCs	peripheral blood mononuclear cells
PBS	phosphate buffered saline
pGREY	peripheral grey matter
RRun	relapsing remitting untreated patients
RS	Rio Score
s.c.	subcutaneous injection
TBV	total brain volume
UPR	unfolded protein response
vCSF	ventricular cerebrospinal fluid
WM	white matter
